# Bis(6-nitro-1,10-phenanthrolin-1-ium) 2,5-di­carb­oxy­terephthalate

**DOI:** 10.1107/S1600536814002414

**Published:** 2014-02-08

**Authors:** Kai-Long Zhong, Chao Ni

**Affiliations:** a Department of Applied Chemistry, Nanjing College of Chemical Technology, Nanjing 210048, People’s Republic of China

## Abstract

In the structure of the title 2:1 proton-transfer compound, 2C_12_H_8_N_3_O_2_^+^·C_10_H_4_O_8_^2−^, the 6-nitro-1,10-phenanthroline mol­ecules act as proton sponges, accepting protons from pyromellitic acid. The –NO_2_ group of one of the 6-nitro-1,10-phenanthrolin-1-ium cations is disordered and was refined with a site-occupancy ratio of 0.624 (15):0.376 (15). Two –COOH(–COO^−^) groups of the 2,5-di­carb­oxy­terephthalate dianion are disordered and were refined with site-occupancy ratios of 0.769 (4):0.231 (4) and 0.766 (5):0.234 (5). The –NO_2_ group of the second cation is also disordered about a pseudo-twofold rotation axis and was refined with a site-occupancy ratio of 0.903 (3):0.097 (3). There is an intra­molecular O—H⋯O hydrogen bond in the anion. The phenanthroline rings of the two cations are inclined to one another by 31.3 (1)°. In the anions, considering the major components only, the carb­oxy­lic acid groups (–COOH) are inclined to the benzene ring by 17.3 (2) and 22.3 (3)°. The carboxyl­ate groups (–COO^−^) are twisted by 9.3 (2) and 13.6 (6)° with respect to the benzene ring. In the crystal, adjacent 2,5-di­carb­oxy­terephthalate anions are linked *via* O—H⋯O hydrogen bonds, forming chains propagating along [010]. The cations are attached to the chain of anions by N—H⋯O hydrogen bonds.

## Related literature   

For related structures involving pyromellitic acid, see: Li *et al.* (2003 ▶); Guo *et al.* (2007 ▶); Fabelo *et al.* (2008 ▶); Zhong (2013 ▶).
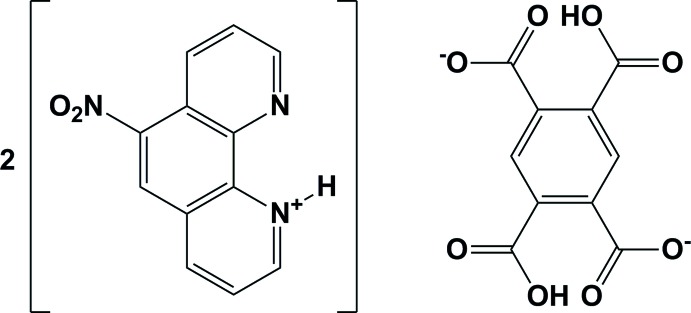


## Experimental   

### 

#### Crystal data  

2C_12_H_8_N_3_O_2_^+^·C_10_H_4_O_8_^2−^
*M* *_r_* = 704.56
Triclinic, 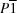

*a* = 8.5937 (6) Å
*b* = 9.8302 (7) Å
*c* = 18.8700 (14) Å
α = 77.810 (2)°
β = 83.622 (2)°
γ = 68.025 (2)°
*V* = 1444.05 (18) Å^3^
*Z* = 2
Mo *K*α radiation
μ = 0.13 mm^−1^
*T* = 223 K
0.25 × 0.20 × 0.15 mm

#### Data collection  

Rigaku Mercury CCD diffractometer
Absorption correction: multi-scan (*REQAB*; Jacobson, 1998 ▶) *T*_min_ = 0.969, *T*_max_ = 0.981
30563 measured reflections
5237 independent reflections
3942 reflections with *I* > 2σ(*I*)
*R*_int_ = 0.036

#### Refinement  

*R*[*F* ^2^ > 2σ(*F* ^2^)] = 0.068
*wR*(*F* ^2^) = 0.191
*S* = 1.05
5237 reflections
518 parameters
24 restraints
H-atom parameters constrained
Δρ_max_ = 0.72 e Å^−3^
Δρ_min_ = −0.73 e Å^−3^

### 

Data collection: *CrystalClear* (Rigaku, 2007 ▶); cell refinement: *CrystalClear*; data reduction: *CrystalClear*; program(s) used to solve structure: *SHELXS97* (Sheldrick, 2008 ▶); program(s) used to refine structure: *SHELXL97* (Sheldrick, 2008 ▶); molecular graphics: *XP* in *SHELXTL* (Sheldrick, 2008 ▶); software used to prepare material for publication: *SHELXTL*.

## Supplementary Material

Crystal structure: contains datablock(s) global, I. DOI: 10.1107/S1600536814002414/su2687sup1.cif

Structure factors: contains datablock(s) I. DOI: 10.1107/S1600536814002414/su2687Isup2.hkl

Supporting information file. DOI: 10.1107/S1600536814002414/su2687Isup3.cml

CCDC reference:

Additional supporting information:  crystallographic information; 3D view; checkCIF report

## Figures and Tables

**Table 1:** Hydrogen-bond geometry (Å, °)

*D*—H⋯*A*	*D*—H	H⋯*A*	*D*⋯*A*	*D*—H⋯*A*
O6—H6*O*⋯O8	0.83	1.36	2.031 (10)	135
O6—H6*O*⋯O8′	0.83	2.50	3.024 (4)	123
O10—H10*O*⋯O5^i^	0.83	1.92	2.724 (4)	164
N2—H2*N*′⋯O7′^ii^	0.87	2.16	3.012 (4)	165
N3—H3*N*⋯O7′^ii^	0.87	1.86	2.695 (4)	160
N2—H2*N*′⋯O7^ii^	0.87	2.21	3.078 (9)	172
N3—H3*N*⋯O7^ii^	0.87	1.84	2.678 (11)	162
N6—H6*N*⋯O11^iii^	0.87	1.85	2.712 (4)	168

Symmetry codes: (i) 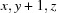; (ii) 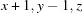; (iii) 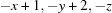.

**Fig. 1.**
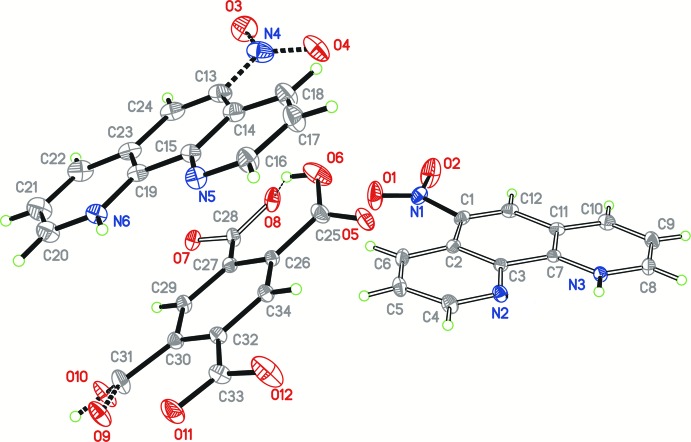
The molecular structure of the title compound, with atom labelling. Displacement ellipsoids are drawn at the 30% probability level. Only the major components of the disordered atoms are shown. There is an intramolecular O—H···O hydrogen bond in the anion shown as a thin dashed line (See Table 1 for details).

**Fig. 2.**
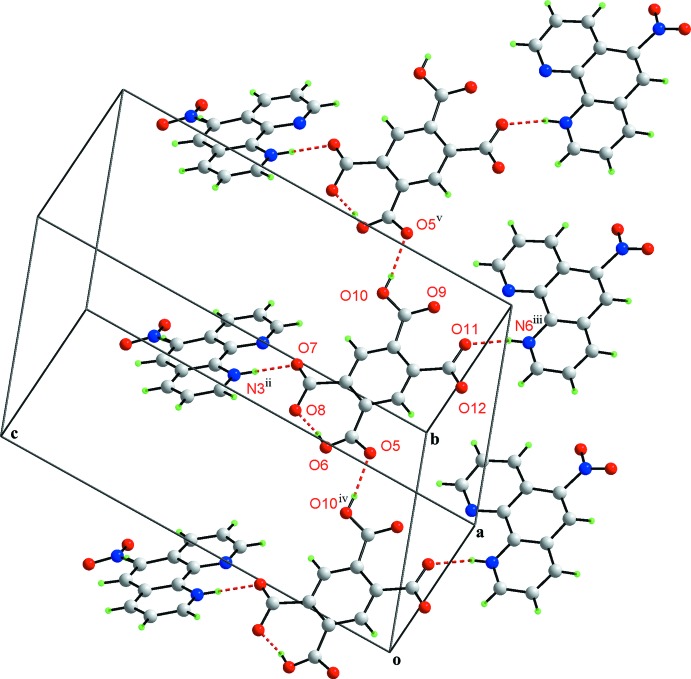
A view along the a axis of the crystal packing of the title compound. The hydrogen bonds are shown as dashed lines (see Table 1 for details; symmetry codes: (i) x, y + 1, z; (ii) x + 1, y - 1, z; (iii) -x + 1, -y + 2, -z; (iv) x, - y + 1, z; (v) x, y + 1, z].

## supplementary crystallographic information

### 1. Comment

Pyromellitic (PMA) acid (Li *et al.*, 2003; Fabelo *et al.*, 2008) has been widely use in constructing interesting supramolecular networks because it can act not only as an hydrogen bond acceptor but also as an hydrogen bond donor, depending upon the number of deprotonated carboxylate groups present. Proton-transfer compounds of PMA with 1,10-phenanthroline for example 1,10-Phenanthrolinium trihydrogen-1,2,4,5-benzenetetracarboxylate monohydrate [Guo *et al.*, 2007] and a substituted phenanthroline 2,9-Dimethyl-1,10-phenanthrolin-1-ium 2,4,5-tricarboxybenzoate monohydrate [Zhong, 2013] have been synthesized and reported. The title compound was obtained using PMA and 6-nitro-1,10-phenanthroline, *via* a thermal reaction and we report herein on its crystal structure.

The asymmetric unit of the title compound consists of two 6-nitro-1,10-phenanthrolin-1-ium cations, one 2,5-dicarboxyterephthalate anion, Fig. 1. The proton transfer is from two carboxyl groups to the ring N atoms (N3 and N6) of the 5-nitro-1,10-phenanthroline cations. In the anion, the dihedral angles between the benzene ring of PMA^2-^ and the mean-planes of the COOH (COO^-^) groups are 17.3 (2) ° for (O5/C25/O6), 22.3 (3) ° for (O9/C31/O10), 13.6 (6) ° for (O7/C28/O8) and 9.3 (2)° for (O11/C33/O12). An intramolecular O—H···O hydrogen bond is observed in the anion (Fig. 1 and Table 1).

In the crystal, adjacent PMA^2-^ anions interact *via* O—H···O hydrogen bonds, forming one-dimensional chains along the *b* axis, and the 6-nitro-1,10-phenanthrolin-1-ium cations are attached to the PMA^2-^ anions by N—H···O hydrogen bonds (Fig. 2 and Table 1).

### 2. Experimental

0.1 mmol 5-nitro-1,10-phenanthroline, 0.1 mmol 1,2,4,5-benzenetetracarboxylic acid, and 2.0 ml water were mixed and placed in a thick Pyrex tube, which was sealed and heated to 383 K for 72 h, whereupon pink block-shaped crystals of the title compound were obtained.

### 3. Refinement

The NH H atoms and the carboxyl H atoms were located from difference electron-density maps. In the final cycles of refinement they were included in calculated positions and treated as riding atoms: N—H = 0.87 Å and O—H = 0.83 Å with*U*_iso_(H) = 1.2*U*_eq_(N) and = 1.5*U*_eq_(O). The C bound H atoms were positioned geometrically and allowed to ride on their parent atoms: C—H = 0.94 Å with *U*_iso_(H) = 1.2*U*_eq_(C). The –NO_2_ group (atoms O3/O4) of the one of the 5-nitro-1,10-phenanthrolin-1-ium cations was refined with a site-occupancy ratio of 0.624 (15):0.376 (15). Two –COOH(–COO^-^) groups (atoms O7/O8 and O9) of the 2,5-dicarboxyterephthalate dianion are disordered and were refined with site-occupancy ratios of 0.769 (4):0.231 (4) and 0.766 (5):0.234 (5), respectively. The –NO_2_ group (atoms N1/O1/O2) of the second cation is also disordered about a pseudo twofold rotation axis and was refined with a site-occupancy ratio of 0.903 (3):0.097 (3).

### Crystal data

**Table:**

2C_12_H_8_N_3_O_2_^+^·C_10_H_4_O_8_^2^^−^	*Z* = 2
*M**_r_* = 704.56	*F*(000) = 724
Triclinic, *P*1	*D*_x_ = 1.620 Mg m^−^^3^
Hall symbol: -P 1	Mo *K*α radiation, λ = 0.71073 Å
*a* = 8.5937 (6) Å	Cell parameters from 9974 reflections
*b* = 9.8302 (7) Å	θ = 2.3–27.2°
*c* = 18.8700 (14) Å	µ = 0.13 mm^−^^1^
α = 77.810 (2)°	*T* = 223 K
β = 83.622 (2)°	Block, pink
γ = 68.025 (2)°	0.25 × 0.20 × 0.15 mm
*V* = 1444.05 (18) Å^3^	

### Data collection

**Table:**

Rigaku Mercury CCD diffractometer	5237 independent reflections
Radiation source: fine-focus sealed tube	3942 reflections with *I* > 2σ(*I*)
Graphite Monochromator monochromator	*R*_int_ = 0.036
Detector resolution: 28.5714 pixels mm^-1^	θ_max_ = 25.3°, θ_min_ = 2.3°
ω scans	*h* = −10→10
Absorption correction: multi-scan (*REQAB*; Jacobson, 1998)	*k* = −11→11
*T*_min_ = 0.969, *T*_max_ = 0.981	*l* = −22→22
30563 measured reflections	

### Refinement

**Table:**

Refinement on *F*^2^	Primary atom site location: structure-invariant direct methods
Least-squares matrix: full	Secondary atom site location: difference Fourier map
*R*[*F*^2^ > 2σ(*F*^2^)] = 0.068	Hydrogen site location: inferred from neighbouring sites
*wR*(*F*^2^) = 0.191	H-atom parameters constrained
*S* = 1.05	*w* = 1/[σ^2^(*F*_o_^2^) + (0.0939*P*)^2^ + 1.9537*P*] where *P* = (*F*_o_^2^ + 2*F*_c_^2^)/3
5237 reflections	(Δ/σ)_max_ = 0.001
518 parameters	Δρ_max_ = 0.72 e Å^−^^3^
24 restraints	Δρ_min_ = −0.73 e Å^−^^3^

### Special details

**Table:**

Geometry. All e.s.d.'s (except the e.s.d. in the dihedral angle between two l.s. planes) are estimated using the full covariance matrix. The cell e.s.d.'s are taken into account individually in the estimation of e.s.d.'s in distances, angles and torsion angles; correlations between e.s.d.'s in cell parameters are only used when they are defined by crystal symmetry. An approximate (isotropic) treatment of cell e.s.d.'s is used for estimating e.s.d.'s involving l.s. planes.
Refinement. Refinement of *F*^2^ against ALL reflections. The weighted *R*-factor *wR* and goodness of fit *S* are based on *F*^2^, conventional *R*-factors *R* are based on *F*, with *F* set to zero for negative *F*^2^. The threshold expression of *F*^2^ > σ(*F*^2^) is used only for calculating *R*-factors(gt) *etc*. and is not relevant to the choice of reflections for refinement. *R*-factors based on *F*^2^ are statistically about twice as large as those based on *F*, and *R*- factors based on ALL data will be even larger.

### Fractional atomic coordinates and isotropic or equivalent isotropic displacement parameters (Å^2^)

**Table:**

	*x*	*y*	*z*	*U*_iso_*/*U*_eq_	Occ. (<1)
O1	0.3137 (4)	0.3400 (3)	0.5554 (2)	0.0744 (12)	0.903 (3)
O1'	0.491 (4)	−0.164 (2)	0.6658 (19)	0.0744 (12)	0.097 (3)
O2	0.3275 (5)	0.1593 (4)	0.64151 (19)	0.0756 (12)	0.903 (3)
O2'	0.381 (4)	0.069 (3)	0.6705 (19)	0.0756 (12)	0.097 (3)
O3	−0.1801 (12)	0.5821 (17)	0.2706 (6)	0.068 (3)	0.624 (15)
O3'	−0.123 (3)	0.582 (3)	0.2876 (10)	0.094 (7)	0.376 (15)
O4	0.0292 (11)	0.4192 (7)	0.2234 (3)	0.065 (3)	0.624 (15)
O4'	−0.0960 (19)	0.4854 (14)	0.1923 (7)	0.082 (5)	0.376 (15)
O5	0.5115 (3)	0.4118 (3)	0.20188 (16)	0.0523 (7)	
O6	0.2903 (4)	0.4939 (3)	0.27486 (15)	0.0641 (9)	
H6O	0.2334	0.5721	0.2898	0.096*	
O7'	0.1076 (4)	0.8482 (3)	0.33626 (16)	0.0324 (8)	0.769 (4)
O7	0.1737 (14)	0.8568 (10)	0.3577 (6)	0.0324 (8)	0.231 (4)
O8'	0.3302 (4)	0.6580 (3)	0.38628 (16)	0.0420 (8)	0.769 (4)
O8	0.2274 (13)	0.6234 (9)	0.3520 (5)	0.0420 (8)	0.231 (4)
O9	0.5320 (5)	1.1519 (3)	0.11200 (17)	0.0479 (11)	0.766 (5)
O9'	0.3760 (13)	1.1813 (10)	0.1143 (5)	0.045 (3)	0.234 (5)
O10	0.4440 (6)	1.1561 (4)	0.22447 (18)	0.0537 (11)	0.766 (5)
H10O	0.4491	1.2406	0.2125	0.081*	0.766 (5)
O10'	0.5481 (17)	1.1344 (13)	0.2056 (6)	0.0537 (11)	0.234 (5)
H10'	0.5945	1.0636	0.2383	0.081*	0.234 (5)
O11	0.6743 (3)	0.9654 (3)	0.04547 (15)	0.0536 (7)	
O12	0.7415 (5)	0.7292 (3)	0.04701 (17)	0.0751 (11)	
N1	0.3874 (4)	0.2155 (4)	0.58681 (16)	0.0355 (8)	0.903 (3)
N1'	0.475 (2)	−0.0347 (19)	0.6420 (9)	0.0355 (8)	0.097 (3)
H12A	0.5463	−0.0502	0.6377	0.043*	0.903 (3)
N2	0.8495 (3)	0.1032 (3)	0.39818 (13)	0.0283 (6)	
H2N'	0.9372	0.0357	0.3824	0.034*	0.097 (3)
N3	0.9666 (3)	−0.1817 (3)	0.47071 (13)	0.0245 (6)	
H3N	1.0141	−0.1538	0.4300	0.029*	0.903 (3)
N4	−0.0665 (5)	0.5579 (4)	0.2275 (2)	0.0570 (9)	
N5	0.2343 (4)	0.7762 (3)	0.02188 (14)	0.0364 (7)	
N6	0.1136 (3)	1.0338 (3)	0.07212 (14)	0.0336 (6)	
H6N	0.1811	1.0217	0.0341	0.040*	
C1	0.5424 (4)	0.1202 (4)	0.55391 (17)	0.0293 (7)	
H1A	0.4448	0.1871	0.5728	0.035*	0.097 (3)
C2	0.6201 (4)	0.1748 (3)	0.48754 (16)	0.0258 (7)	
C3	0.7642 (4)	0.0698 (3)	0.45973 (15)	0.0231 (6)	
C4	0.7944 (4)	0.2422 (3)	0.36302 (17)	0.0334 (7)	
H4	0.8522	0.2669	0.3199	0.040*	
C5	0.6551 (4)	0.3547 (3)	0.38622 (18)	0.0355 (8)	
H5	0.6222	0.4526	0.3596	0.043*	
C6	0.5674 (4)	0.3217 (3)	0.44759 (18)	0.0330 (7)	
H6	0.4723	0.3964	0.4633	0.040*	
C7	0.8291 (3)	−0.0827 (3)	0.49830 (15)	0.0222 (6)	
C8	1.0286 (4)	−0.3212 (3)	0.50596 (17)	0.0299 (7)	
H8	1.1248	−0.3884	0.4863	0.036*	
C9	0.9570 (4)	−0.3723 (4)	0.57085 (18)	0.0346 (8)	
H9	1.0041	−0.4721	0.5944	0.042*	
C10	0.8179 (4)	−0.2759 (4)	0.59978 (17)	0.0327 (7)	
H10	0.7677	−0.3083	0.6436	0.039*	
C11	0.7504 (4)	−0.1275 (3)	0.56345 (16)	0.0261 (7)	
C12	0.6032 (4)	−0.0210 (4)	0.58953 (17)	0.0307 (7)	
C13	−0.0143 (5)	0.6806 (4)	0.18440 (19)	0.0414 (9)	
C14	0.0943 (4)	0.6569 (4)	0.12056 (18)	0.0389 (8)	
C15	0.1353 (4)	0.7798 (4)	0.08272 (17)	0.0315 (7)	
C16	0.2957 (5)	0.6494 (4)	−0.0025 (2)	0.0450 (9)	
H16	0.3643	0.6450	−0.0451	0.054*	
C17	0.2641 (6)	0.5225 (4)	0.0315 (2)	0.0543 (10)	
H17	0.3117	0.4345	0.0122	0.065*	
C18	0.1644 (5)	0.5253 (4)	0.0926 (2)	0.0512 (10)	
H18	0.1428	0.4393	0.1160	0.061*	
C19	0.0706 (4)	0.9189 (4)	0.10846 (16)	0.0308 (7)	
C20	0.0556 (5)	1.1644 (4)	0.0928 (2)	0.0434 (9)	
H20	0.0873	1.2428	0.0661	0.052*	
C21	−0.0522 (5)	1.1882 (5)	0.1538 (2)	0.0524 (10)	
H21	−0.0954	1.2823	0.1675	0.063*	
C22	−0.0935 (5)	1.0725 (5)	0.1930 (2)	0.0469 (9)	
H22	−0.1623	1.0857	0.2352	0.056*	
C23	−0.0344 (4)	0.9347 (4)	0.17093 (17)	0.0360 (8)	
C24	−0.0752 (4)	0.8099 (4)	0.20862 (18)	0.0411 (9)	
H24	−0.1452	0.8184	0.2507	0.049*	
C25	0.4054 (5)	0.5133 (4)	0.23522 (19)	0.0401 (9)	
C26	0.4305 (4)	0.6590 (3)	0.21941 (16)	0.0254 (6)	
C27	0.3503 (4)	0.7690 (3)	0.26201 (16)	0.0251 (6)	
C28	0.2554 (4)	0.7475 (3)	0.33295 (16)	0.0299 (7)	
C29	0.3697 (4)	0.9063 (3)	0.24121 (16)	0.0283 (7)	
H29	0.3145	0.9802	0.2693	0.034*	
C30	0.4667 (4)	0.9397 (3)	0.18090 (16)	0.0274 (7)	
C31	0.4709 (5)	1.0956 (4)	0.16873 (18)	0.0449 (10)	
C32	0.5472 (4)	0.8295 (3)	0.13834 (16)	0.0273 (7)	
C33	0.6613 (4)	0.8400 (4)	0.07175 (17)	0.0340 (8)	
C34	0.5260 (4)	0.6923 (3)	0.15919 (16)	0.0268 (7)	
H34	0.5796	0.6187	0.1307	0.032*	

### Atomic displacement parameters (Å^2^)

**Table:**

	*U*^11^	*U*^22^	*U*^33^	*U*^12^	*U*^13^	*U*^23^
O1	0.064 (2)	0.0355 (18)	0.093 (3)	0.0035 (16)	0.0379 (19)	−0.0064 (17)
O1'	0.064 (2)	0.0355 (18)	0.093 (3)	0.0035 (16)	0.0379 (19)	−0.0064 (17)
O2	0.065 (2)	0.070 (3)	0.057 (2)	0.0023 (19)	0.0344 (18)	−0.0056 (18)
O2'	0.065 (2)	0.070 (3)	0.057 (2)	0.0023 (19)	0.0344 (18)	−0.0056 (18)
O3	0.059 (5)	0.075 (4)	0.063 (5)	−0.036 (5)	0.018 (4)	0.015 (4)
O3'	0.090 (14)	0.104 (10)	0.071 (11)	−0.043 (12)	0.026 (9)	0.020 (9)
O4	0.102 (6)	0.051 (4)	0.058 (3)	−0.048 (4)	0.003 (3)	−0.006 (3)
O4'	0.096 (11)	0.066 (7)	0.106 (9)	−0.056 (8)	0.017 (8)	−0.020 (7)
O5	0.0588 (17)	0.0277 (13)	0.0744 (19)	−0.0193 (12)	0.0006 (14)	−0.0126 (13)
O6	0.093 (2)	0.070 (2)	0.0496 (17)	−0.0616 (19)	0.0145 (16)	−0.0015 (14)
O7'	0.0319 (19)	0.0326 (14)	0.0226 (17)	−0.0022 (14)	0.0039 (13)	−0.0037 (12)
O7	0.0319 (19)	0.0326 (14)	0.0226 (17)	−0.0022 (14)	0.0039 (13)	−0.0037 (12)
O8'	0.0454 (19)	0.0321 (16)	0.0299 (16)	0.0002 (13)	0.0076 (13)	0.0028 (13)
O8	0.0454 (19)	0.0321 (16)	0.0299 (16)	0.0002 (13)	0.0076 (13)	0.0028 (13)
O9	0.075 (3)	0.0351 (18)	0.0397 (19)	−0.0317 (18)	0.0217 (17)	−0.0100 (14)
O9'	0.053 (8)	0.029 (5)	0.057 (7)	−0.024 (5)	−0.009 (5)	0.005 (5)
O10	0.094 (4)	0.0337 (17)	0.045 (2)	−0.040 (2)	0.0231 (19)	−0.0130 (15)
O10'	0.094 (4)	0.0337 (17)	0.045 (2)	−0.040 (2)	0.0231 (19)	−0.0130 (15)
O11	0.0618 (18)	0.0396 (15)	0.0605 (17)	−0.0292 (13)	0.0254 (14)	−0.0053 (13)
O12	0.109 (3)	0.064 (2)	0.073 (2)	−0.0548 (19)	0.0611 (19)	−0.0440 (17)
N1	0.0292 (17)	0.0409 (19)	0.0323 (17)	−0.0057 (14)	0.0047 (13)	−0.0140 (14)
N1'	0.0292 (17)	0.0409 (19)	0.0323 (17)	−0.0057 (14)	0.0047 (13)	−0.0140 (14)
N2	0.0332 (15)	0.0240 (13)	0.0267 (13)	−0.0109 (11)	0.0042 (11)	−0.0044 (11)
N3	0.0264 (13)	0.0217 (13)	0.0247 (13)	−0.0092 (10)	0.0057 (10)	−0.0049 (10)
N4	0.066 (3)	0.071 (3)	0.046 (2)	−0.044 (2)	0.0018 (18)	0.0004 (18)
N5	0.0405 (17)	0.0386 (16)	0.0285 (14)	−0.0132 (13)	0.0065 (12)	−0.0088 (12)
N6	0.0363 (15)	0.0400 (16)	0.0277 (14)	−0.0168 (13)	0.0070 (11)	−0.0116 (12)
C1	0.0217 (16)	0.0374 (18)	0.0308 (17)	−0.0081 (14)	0.0014 (13)	−0.0164 (14)
C2	0.0225 (15)	0.0272 (16)	0.0289 (16)	−0.0068 (12)	−0.0027 (12)	−0.0110 (13)
C3	0.0227 (15)	0.0238 (15)	0.0241 (15)	−0.0098 (12)	−0.0003 (12)	−0.0052 (12)
C4	0.044 (2)	0.0289 (17)	0.0267 (16)	−0.0151 (15)	0.0006 (14)	−0.0007 (13)
C5	0.043 (2)	0.0213 (16)	0.0372 (18)	−0.0068 (14)	−0.0065 (15)	−0.0010 (13)
C6	0.0302 (17)	0.0253 (16)	0.0383 (18)	−0.0014 (13)	−0.0043 (14)	−0.0097 (14)
C7	0.0208 (15)	0.0248 (15)	0.0225 (14)	−0.0093 (12)	−0.0005 (11)	−0.0060 (12)
C8	0.0297 (17)	0.0232 (15)	0.0335 (17)	−0.0054 (13)	−0.0009 (13)	−0.0061 (13)
C9	0.0413 (19)	0.0237 (16)	0.0364 (18)	−0.0120 (14)	−0.0037 (15)	0.0016 (13)
C10	0.0381 (19)	0.0339 (18)	0.0265 (16)	−0.0182 (15)	0.0023 (13)	0.0018 (14)
C11	0.0259 (16)	0.0313 (16)	0.0246 (15)	−0.0145 (13)	0.0013 (12)	−0.0062 (12)
C12	0.0281 (17)	0.0405 (19)	0.0277 (16)	−0.0164 (14)	0.0066 (13)	−0.0113 (14)
C13	0.043 (2)	0.055 (2)	0.0313 (18)	−0.0286 (18)	−0.0008 (15)	0.0009 (16)
C14	0.041 (2)	0.045 (2)	0.0332 (18)	−0.0209 (17)	−0.0028 (15)	−0.0015 (15)
C15	0.0331 (18)	0.0369 (18)	0.0263 (16)	−0.0156 (14)	0.0014 (13)	−0.0055 (13)
C16	0.052 (2)	0.039 (2)	0.043 (2)	−0.0147 (17)	0.0113 (17)	−0.0165 (17)
C17	0.066 (3)	0.038 (2)	0.061 (3)	−0.0191 (19)	0.011 (2)	−0.0184 (19)
C18	0.064 (3)	0.038 (2)	0.056 (2)	−0.0261 (19)	0.007 (2)	−0.0073 (18)
C19	0.0302 (17)	0.0432 (19)	0.0221 (15)	−0.0161 (15)	−0.0002 (13)	−0.0075 (14)
C20	0.047 (2)	0.044 (2)	0.045 (2)	−0.0211 (17)	0.0122 (17)	−0.0201 (17)
C21	0.057 (3)	0.055 (2)	0.054 (2)	−0.023 (2)	0.0160 (19)	−0.031 (2)
C22	0.044 (2)	0.066 (3)	0.038 (2)	−0.0224 (19)	0.0148 (16)	−0.0285 (19)
C23	0.0324 (18)	0.055 (2)	0.0256 (16)	−0.0192 (16)	0.0024 (13)	−0.0133 (15)
C24	0.0369 (19)	0.066 (3)	0.0248 (17)	−0.0262 (18)	0.0030 (14)	−0.0050 (16)
C25	0.055 (2)	0.0309 (18)	0.0377 (19)	−0.0223 (17)	−0.0136 (17)	0.0062 (15)
C26	0.0263 (16)	0.0225 (15)	0.0263 (15)	−0.0094 (12)	−0.0037 (12)	0.0004 (12)
C27	0.0249 (15)	0.0244 (15)	0.0251 (15)	−0.0087 (12)	−0.0012 (12)	−0.0026 (12)
C28	0.0340 (18)	0.0208 (15)	0.0310 (17)	−0.0074 (14)	0.0059 (14)	−0.0047 (13)
C29	0.0324 (17)	0.0255 (16)	0.0253 (16)	−0.0086 (13)	0.0032 (13)	−0.0066 (12)
C30	0.0292 (16)	0.0250 (16)	0.0271 (16)	−0.0101 (13)	0.0007 (12)	−0.0030 (12)
C31	0.054 (2)	0.0260 (17)	0.049 (2)	−0.0144 (17)	0.0295 (19)	−0.0101 (16)
C32	0.0310 (17)	0.0283 (16)	0.0240 (15)	−0.0129 (13)	0.0012 (12)	−0.0050 (12)
C33	0.042 (2)	0.0395 (19)	0.0274 (17)	−0.0221 (16)	0.0069 (14)	−0.0117 (15)
C34	0.0313 (17)	0.0247 (15)	0.0253 (15)	−0.0101 (13)	0.0004 (13)	−0.0069 (12)

### Geometric parameters (Å, º)

**Table:**

O1—N1	1.203 (4)	C4—C5	1.395 (5)
O1'—N1'	1.219 (18)	C4—H4	0.9400
O1'—H12A	1.3632	C5—C6	1.358 (5)
O2—N1	1.220 (4)	C5—H5	0.9400
O2'—N1'	1.223 (19)	C6—H6	0.9400
O3—N4	1.184 (10)	C7—C11	1.405 (4)
O3'—N4	1.218 (17)	C8—C9	1.391 (5)
O4—N4	1.316 (7)	C8—H8	0.9400
O4'—N4	1.177 (10)	C9—C10	1.360 (5)
O5—C25	1.296 (4)	C9—H9	0.9400
O6—C25	1.221 (5)	C10—C11	1.402 (4)
O6—H6O	0.8300	C10—H10	0.9400
O7'—C28	1.290 (4)	C11—C12	1.424 (4)
O7—C28	1.200 (8)	C12—H12A	1.0218
O8'—C28	1.243 (4)	C13—C24	1.339 (5)
O8—C28	1.300 (8)	C13—C14	1.443 (5)
O9—C31	1.253 (4)	C14—C18	1.396 (5)
O9'—C31	1.317 (7)	C14—C15	1.412 (5)
O10—C31	1.273 (4)	C15—C19	1.437 (5)
O10—H10O	0.8300	C16—C17	1.385 (5)
O10'—C31	1.214 (7)	C16—H16	0.9400
O10'—H10'	0.8300	C17—C18	1.358 (6)
O11—C33	1.269 (4)	C17—H17	0.9400
O12—C33	1.209 (4)	C18—H18	0.9400
N1—C1	1.472 (4)	C19—C23	1.402 (4)
N1'—C12	1.423 (16)	C20—C21	1.397 (5)
N1'—H12A	0.5737	C20—H20	0.9400
N2—C4	1.316 (4)	C21—C22	1.362 (6)
N2—C3	1.350 (4)	C21—H21	0.9400
N2—H2N'	0.8700	C22—C23	1.394 (5)
N3—C8	1.324 (4)	C22—H22	0.9400
N3—C7	1.353 (4)	C23—C24	1.425 (5)
N3—H3N	0.8700	C24—H24	0.9400
N4—C13	1.492 (5)	C25—C26	1.491 (4)
N5—C16	1.319 (4)	C26—C34	1.382 (4)
N5—C15	1.349 (4)	C26—C27	1.406 (4)
N6—C20	1.319 (4)	C27—C29	1.390 (4)
N6—C19	1.346 (4)	C27—C28	1.506 (4)
N6—H6N	0.8700	C29—C30	1.391 (4)
C1—C12	1.339 (5)	C29—H29	0.9400
C1—C2	1.449 (4)	C30—C32	1.407 (4)
C1—H1A	0.9400	C30—C31	1.514 (4)
C2—C6	1.409 (4)	C32—C34	1.397 (4)
C2—C3	1.415 (4)	C32—C33	1.516 (4)
C3—C7	1.446 (4)	C34—H34	0.9400
			
C25—O6—H6O	109.5	C18—C14—C13	127.1 (3)
C31—O10—H10O	109.5	C15—C14—C13	116.4 (3)
C31—O10'—H10'	109.5	N5—C15—C14	123.7 (3)
O1—N1—O2	121.6 (3)	N5—C15—C19	116.1 (3)
O1—N1—C1	119.7 (3)	C14—C15—C19	120.2 (3)
O2—N1—C1	118.1 (3)	N5—C16—C17	123.2 (3)
O2'—N1'—O1'	123 (3)	N5—C16—H16	118.4
O2'—N1'—C12	123 (2)	C17—C16—H16	118.4
O1'—N1'—C12	112.6 (19)	C18—C17—C16	120.0 (4)
O2'—N1'—H12A	125.2	C18—C17—H17	120.0
O1'—N1'—H12A	91.8	C16—C17—H17	120.0
C4—N2—C3	117.3 (3)	C17—C18—C14	119.4 (4)
C4—N2—H2N'	121.3	C17—C18—H18	120.3
C3—N2—H2N'	121.3	C14—C18—H18	120.3
C8—N3—C7	119.7 (3)	N6—C19—C23	120.2 (3)
C8—N3—H3N	120.2	N6—C19—C15	119.1 (3)
C7—N3—H3N	120.2	C23—C19—C15	120.7 (3)
O4'—N4—O3	103.7 (8)	N6—C20—C21	121.0 (4)
O4'—N4—O3'	128.7 (14)	N6—C20—H20	119.5
O4'—N4—O4	55.9 (7)	C21—C20—H20	119.5
O3—N4—O4	119.8 (8)	C22—C21—C20	118.8 (4)
O3'—N4—O4	116.5 (14)	C22—C21—H21	120.6
O4'—N4—C13	114.4 (6)	C20—C21—H21	120.6
O3—N4—C13	121.3 (8)	C21—C22—C23	120.5 (3)
O3'—N4—C13	112.0 (14)	C21—C22—H22	119.8
O4—N4—C13	118.4 (4)	C23—C22—H22	119.8
C16—N5—C15	117.2 (3)	C22—C23—C19	117.9 (3)
C20—N6—C19	121.5 (3)	C22—C23—C24	123.4 (3)
C20—N6—H6N	119.3	C19—C23—C24	118.7 (3)
C19—N6—H6N	119.3	C13—C24—C23	120.3 (3)
C12—C1—C2	123.1 (3)	C13—C24—H24	119.8
C12—C1—N1	114.5 (3)	C23—C24—H24	119.8
C2—C1—N1	122.4 (3)	O6—C25—O5	123.2 (3)
C12—C1—H1A	118.5	O6—C25—C26	122.0 (3)
C2—C1—H1A	118.5	O5—C25—C26	114.7 (3)
C6—C2—C3	116.3 (3)	C34—C26—C27	118.3 (3)
C6—C2—C1	126.9 (3)	C34—C26—C25	120.1 (3)
C3—C2—C1	116.8 (3)	C27—C26—C25	121.5 (3)
N2—C3—C2	123.7 (3)	C29—C27—C26	118.5 (3)
N2—C3—C7	116.3 (2)	C29—C27—C28	115.8 (3)
C2—C3—C7	119.9 (3)	C26—C27—C28	125.4 (3)
N2—C4—C5	123.6 (3)	O7—C28—O8'	103.2 (6)
N2—C4—H4	118.2	O8'—C28—O7'	124.4 (3)
C5—C4—H4	118.2	O7—C28—O8	122.1 (7)
C6—C5—C4	119.6 (3)	O8'—C28—O8	62.8 (5)
C6—C5—H5	120.2	O7'—C28—O8	103.2 (5)
C4—C5—H5	120.2	O7—C28—C27	117.5 (6)
C5—C6—C2	119.5 (3)	O8'—C28—C27	120.0 (3)
C5—C6—H6	120.3	O7'—C28—C27	114.0 (3)
C2—C6—H6	120.3	O8—C28—C27	117.3 (5)
N3—C7—C11	120.4 (3)	C27—C29—C30	123.3 (3)
N3—C7—C3	119.1 (3)	C27—C29—H29	118.3
C11—C7—C3	120.5 (3)	C30—C29—H29	118.3
N3—C8—C9	122.6 (3)	C29—C30—C32	118.2 (3)
N3—C8—H8	118.7	C29—C30—C31	114.3 (3)
C9—C8—H8	118.7	C32—C30—C31	127.5 (3)
C10—C9—C8	119.2 (3)	O10'—C31—O9	90.6 (7)
C10—C9—H9	120.4	O9—C31—O10	118.9 (3)
C8—C9—H9	120.4	O10'—C31—O9'	126.0 (8)
C9—C10—C11	119.2 (3)	O9—C31—O9'	58.6 (5)
C9—C10—H10	120.4	O10—C31—O9'	113.0 (6)
C11—C10—H10	120.4	O10'—C31—C30	123.9 (6)
C10—C11—C7	118.9 (3)	O9—C31—C30	122.9 (3)
C10—C11—C12	122.4 (3)	O10—C31—C30	116.5 (3)
C7—C11—C12	118.7 (3)	O9'—C31—C30	110.1 (5)
C1—C12—C11	121.1 (3)	C34—C32—C30	118.2 (3)
C1—C12—N1'	104.9 (8)	C34—C32—C33	114.5 (3)
C11—C12—N1'	132.8 (8)	C30—C32—C33	127.2 (3)
C1—C12—H12A	118.9	O12—C33—O11	122.0 (3)
C11—C12—H12A	119.9	O12—C33—C32	119.7 (3)
C24—C13—C14	123.8 (3)	O11—C33—C32	118.2 (3)
C24—C13—N4	115.9 (3)	C26—C34—C32	123.5 (3)
C14—C13—N4	120.4 (3)	C26—C34—H34	118.3
C18—C14—C15	116.6 (3)	C32—C34—H34	118.3
			
O1—N1—C1—C12	−172.9 (4)	C15—N5—C16—C17	−0.5 (6)
O2—N1—C1—C12	−1.0 (5)	N5—C16—C17—C18	0.6 (7)
O1—N1—C1—C2	5.9 (5)	C16—C17—C18—C14	0.2 (7)
O2—N1—C1—C2	177.7 (3)	C15—C14—C18—C17	−0.9 (6)
C12—C1—C2—C6	−178.3 (3)	C13—C14—C18—C17	179.8 (4)
N1—C1—C2—C6	3.1 (5)	C20—N6—C19—C23	−1.7 (5)
C12—C1—C2—C3	1.6 (4)	C20—N6—C19—C15	178.9 (3)
N1—C1—C2—C3	−177.0 (3)	N5—C15—C19—N6	−1.1 (4)
C4—N2—C3—C2	0.5 (4)	C14—C15—C19—N6	179.0 (3)
C4—N2—C3—C7	−178.7 (3)	N5—C15—C19—C23	179.5 (3)
C6—C2—C3—N2	−0.6 (4)	C14—C15—C19—C23	−0.4 (5)
C1—C2—C3—N2	179.4 (3)	C19—N6—C20—C21	0.5 (5)
C6—C2—C3—C7	178.5 (3)	N6—C20—C21—C22	1.7 (6)
C1—C2—C3—C7	−1.4 (4)	C20—C21—C22—C23	−2.5 (6)
C3—N2—C4—C5	0.4 (5)	C21—C22—C23—C19	1.3 (6)
N2—C4—C5—C6	−1.2 (5)	C21—C22—C23—C24	−178.8 (4)
C4—C5—C6—C2	1.0 (5)	N6—C19—C23—C22	0.8 (5)
C3—C2—C6—C5	−0.2 (4)	C15—C19—C23—C22	−179.8 (3)
C1—C2—C6—C5	179.8 (3)	N6—C19—C23—C24	−179.1 (3)
C8—N3—C7—C11	−1.0 (4)	C15—C19—C23—C24	0.3 (5)
C8—N3—C7—C3	179.2 (3)	C14—C13—C24—C23	0.2 (6)
N2—C3—C7—N3	−1.1 (4)	N4—C13—C24—C23	−179.3 (3)
C2—C3—C7—N3	179.7 (3)	C22—C23—C24—C13	179.9 (3)
N2—C3—C7—C11	179.0 (3)	C19—C23—C24—C13	−0.2 (5)
C2—C3—C7—C11	−0.2 (4)	O6—C25—C26—C34	−160.3 (3)
C7—N3—C8—C9	0.6 (5)	O5—C25—C26—C34	17.6 (4)
N3—C8—C9—C10	−0.1 (5)	O6—C25—C26—C27	15.7 (5)
C8—C9—C10—C11	0.0 (5)	O5—C25—C26—C27	−166.5 (3)
C9—C10—C11—C7	−0.3 (5)	C34—C26—C27—C29	0.5 (4)
C9—C10—C11—C12	178.7 (3)	C25—C26—C27—C29	−175.5 (3)
N3—C7—C11—C10	0.8 (4)	C34—C26—C27—C28	−173.9 (3)
C3—C7—C11—C10	−179.3 (3)	C25—C26—C27—C28	10.1 (5)
N3—C7—C11—C12	−178.2 (3)	C29—C27—C28—O7	15.9 (8)
C3—C7—C11—C12	1.7 (4)	C26—C27—C28—O7	−169.5 (7)
C2—C1—C12—C11	−0.2 (5)	C29—C27—C28—O8'	−110.8 (4)
N1—C1—C12—C11	178.6 (3)	C26—C27—C28—O8'	63.8 (4)
C2—C1—C12—N1'	−169.0 (9)	C29—C27—C28—O7'	55.7 (4)
N1—C1—C12—N1'	9.8 (9)	C26—C27—C28—O7'	−129.7 (3)
C10—C11—C12—C1	179.5 (3)	C29—C27—C28—O8	176.4 (6)
C7—C11—C12—C1	−1.5 (5)	C26—C27—C28—O8	−9.0 (7)
C10—C11—C12—N1'	−15.3 (13)	C26—C27—C29—C30	−1.0 (5)
C7—C11—C12—N1'	163.6 (12)	C28—C27—C29—C30	173.9 (3)
O2'—N1'—C12—C1	−32 (3)	C27—C29—C30—C32	0.9 (5)
O1'—N1'—C12—C1	162 (3)	C27—C29—C30—C31	−179.7 (3)
O2'—N1'—C12—C11	161 (3)	C29—C30—C31—O10'	73.5 (9)
O1'—N1'—C12—C11	−5 (3)	C32—C30—C31—O10'	−107.2 (9)
O4'—N4—C13—C24	140.4 (9)	C29—C30—C31—O9	−169.8 (4)
O3—N4—C13—C24	15.0 (9)	C32—C30—C31—O9	9.6 (6)
O3'—N4—C13—C24	−16.8 (13)	C29—C30—C31—O10	25.4 (5)
O4—N4—C13—C24	−156.7 (5)	C32—C30—C31—O10	−155.2 (4)
O4'—N4—C13—C14	−39.1 (10)	C29—C30—C31—O9'	−104.9 (6)
O3—N4—C13—C14	−164.6 (7)	C32—C30—C31—O9'	74.4 (7)
O3'—N4—C13—C14	163.6 (13)	C29—C30—C32—C34	−0.4 (4)
O4—N4—C13—C14	23.7 (6)	C31—C30—C32—C34	−179.7 (3)
C24—C13—C14—C18	179.0 (4)	C29—C30—C32—C33	−178.5 (3)
N4—C13—C14—C18	−1.5 (6)	C31—C30—C32—C33	2.2 (5)
C24—C13—C14—C15	−0.3 (5)	C34—C32—C33—O12	−9.3 (5)
N4—C13—C14—C15	179.2 (3)	C30—C32—C33—O12	168.9 (4)
C16—N5—C15—C14	−0.4 (5)	C34—C32—C33—O11	173.5 (3)
C16—N5—C15—C19	179.7 (3)	C30—C32—C33—O11	−8.3 (5)
C18—C14—C15—N5	1.1 (5)	C27—C26—C34—C32	0.0 (4)
C13—C14—C15—N5	−179.5 (3)	C25—C26—C34—C32	176.1 (3)
C18—C14—C15—C19	−179.0 (3)	C30—C32—C34—C26	−0.1 (5)
C13—C14—C15—C19	0.4 (5)	C33—C32—C34—C26	178.3 (3)

### Hydrogen-bond geometry (Å, º)

**Table:**

*D*—H···*A*	*D*—H	H···*A*	*D*···*A*	*D*—H···*A*
O6—H6*O*···O8	0.83	1.36	2.031 (10)	135
O6—H6*O*···O8′	0.83	2.50	3.024 (4)	123
O10—H10*O*···O5^i^	0.83	1.92	2.724 (4)	164
N2—H2*N*′···O7′^ii^	0.87	2.16	3.012 (4)	165
N3—H3*N*···O7′^ii^	0.87	1.86	2.695 (4)	160
N2—H2*N*′···O7^ii^	0.87	2.21	3.078 (9)	172
N3—H3*N*···O7^ii^	0.87	1.84	2.678 (11)	162
N6—H6*N*···O11^iii^	0.87	1.85	2.712 (4)	168

Symmetry codes: (i) *x*, *y*+1, *z*; (ii) *x*+1, *y*−1, *z*; (iii) −*x*+1, −*y*+2, −*z*.
